# Rapid control and feedback rates enhance neuroprosthetic control

**DOI:** 10.1038/ncomms13825

**Published:** 2017-01-06

**Authors:** Maryam M. Shanechi, Amy L. Orsborn, Helene G. Moorman, Suraj Gowda, Siddharth Dangi, Jose M. Carmena

**Affiliations:** 1 Department of Electrical Engineering, Viterbi School of Engineering, University of Southern California, Los Angeles, California 90089, USA; 2 Department of Electrical Engineering and Computer Sciences, University of California, Berkeley, Berkeley, California 94720, USA; 3 UC Berkeley — UCSF Joint Graduate Program in Bioengineering, University of California, Berkeley, Berkeley, California 94720, USA; 4 Helen Wills Neuroscience Institute, University of California, Berkeley, Berkeley, California 94720, USA

## Abstract

Brain-machine interfaces (BMI) create novel sensorimotor pathways for action. Much as the sensorimotor apparatus shapes natural motor control, the BMI pathway characteristics may also influence neuroprosthetic control. Here, we explore the influence of control and feedback rates, where control rate indicates how often motor commands are sent from the brain to the prosthetic, and feedback rate indicates how often visual feedback of the prosthetic is provided to the subject. We developed a new BMI that allows arbitrarily fast control and feedback rates, and used it to dissociate the effects of each rate in two monkeys. Increasing the control rate significantly improved control even when feedback rate was unchanged. Increasing the feedback rate further facilitated control. We also show that our high-rate BMI significantly outperformed state-of-the-art methods due to higher control and feedback rates, combined with a different point process mathematical encoding model. Our BMI paradigm can dissect the contribution of different elements in the sensorimotor pathway, providing a unique tool for studying neuroprosthetic control mechanisms.

## Abstract (web summary)

Brain machine interfaces (BMI) enable sensorimotor control of movement yet the parameters that may affect these pathways are not known. Here the authors show systematically that increasing the rate of control from brain as well as feedback rates to the subject results in better performance on a BMI task in monkeys.

Brain-machine interfaces (BMIs) enable the brain to directly control external devices by bypassing natural motor pathways^1^,^2^,^3^,^4^,^5^,^6^,^7^,^8^,^9^,^10^,^11^,^12^,^13^,^14^,^15^,^16^,^17^,^18^,^19^,^20^. BMIs create a bidirectional pathway between the brain and the prosthetic (Fig. 1a,b). In the forward path, the brain sends motor commands to the prosthetic device; and in the feedback path, the user receives visual feedback of the prosthetic response to this motor command (for example, the user watches the BMI output on a computer screen).

Significant work in BMI design in recent years yielded marked improvements in performance. For the last few years, the field converged towards a common approach based on processing spike counts using a Kalman filter (KF) trained in closed-loop operation^13^,^15^,^16^,^19^,^21^. Since the development of closed-loop KF-BMIs, however, new approaches have not been able to significantly improve BMI performance beyond that of KF-BMIs^22^. This plateau calls for a design shift based on a more mechanistic understanding of neuroprosthetic control. BMIs create a novel sensorimotor pathway whose properties may influence control. Investigating these influences can lead not only to a deeper understanding of control strategies in BMIs, but also to new designs that optimize the sensorimotor pathway for enhanced neuroprosthetic control.

In natural motor control, the properties of the sensorimotor apparatus strongly influence neural mechanisms^23^,^24^. Growing research suggests that neuroprosthetic control is also shaped by properties of the BMI system like the dynamics of the controlled device^3^,^25^ and the mapping between neural activity and movement^9^,^19^,^26^,^27^ (the ‘encoding model'). Characteristics of the sensorimotor pathway such as temporal delays^28^ may also have a significant role in neuroprosthetic performance. How the full sensorimotor pathway properties affect neuroprosthetic control, independent of other factors like the encoding model, has not been fully explored. Two fundamental properties of a sensorimotor pathway, distinct from delays, are the control and feedback rates that it enables. One largely unexplored question in BMI is whether these pathway rates influence performance and control strategy.

Understanding how the sensorimotor pathway influences control requires experimental manipulation. BMI paradigms are ideally suited for perturbation experiments because they provide a novel, experimenter-defined sensorimotor pathway^29^,^30^. In contrast to the natural motor system, where control rates may not be easy to manipulate, BMI can distinguish the roles of control and feedback rates in the sensorimotor loop. However, a carefully designed BMI that can disentangle and investigate control and feedback rates has not yet been devised, and experiments to tease apart the effects of each rate separate from other factors have not yet been conducted. Moreover, control and feedback rates will likely influence strategies for neuroprosthetic control and must be carefully considered during BMI design. If increased feedback and control rates enhance performance, this would warrant rate-independent BMIs that can run at arbitrarily fast rates in contrast to the KF-BMIs, which are rate-dependent (see Supplementary Notes 1 and 2). Finally, designing such rate-independent BMIs combined with pathway perturbations may in turn help elucidate the control mechanisms exploited by the brain during neuroprosthetic control.

Towards this goal, in the first part of this work, we design a closed-loop point process filter (PPF) BMI platform to investigate the effects of feedback and control rates on the brain's control of continuous BMI movement. In the second part, we use this platform to propose a BMI that markedly enhances control beyond the current state-of-the-art closed-loop KF-BMIs. Our platform enables neuroprosthetic control at arbitrarily fast rates unlike spike count-based BMIs like the KF. Critically, it also separates rate manipulations from the decoder's mathematical encoding model to dissect influences of each sensorimotor loop element on control (see ‘Methods' section and Supplementary Note 1). The PPF can run at different rates while keeping the encoding model parameters unchanged, allowing for investigation of rate effects without confounding the results with any changes in the encoding model (Supplementary Note 1).

We use this platform to dissociate the effects of feedback and control rates on neuroprosthetic control in two monkeys performing a variety of BMI tasks using neural signals from their motor cortices. We find that increasing the control rate, even with a slower feedback rate, improves control. Increasing the feedback rate also enhances control. On the basis of these findings, we develop a high-rate closed-loop PPF BMI. We demonstrate that this high-rate PPF BMI markedly improves control over the state-of-the-art KF-BMI. We also identify the elements within the BMI system—feedback rate, control rate and point process encoding model—that contribute to these improvements.

## Results

### Experimental manipulations and methodology

**Figure 1:**
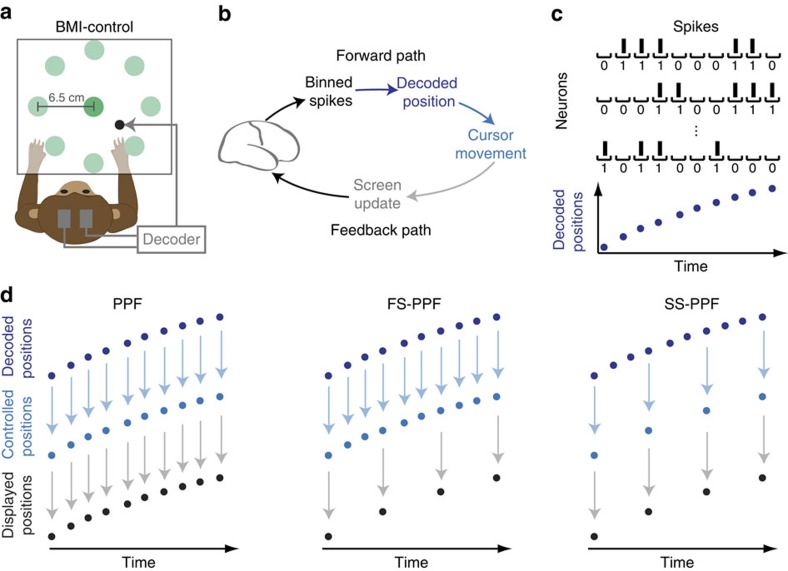
Task design and experimental setup. (**a**) Monkey J performing the self-paced delayed centre-out movement task in brain control. The BMI paradigm introduces a novel bidirectional pathway between the brain and the prosthetic. (**b**) The BMI control and feedback loop, with all pathway processes illustrated. As further illustrated in **d**, control rate was manipulated by modifying the decoded position to cursor movement portion of the loop, while feedback rate was manipulated by adjusting the cursor movement to screen-update portion of the loop. (**c**) Each row corresponds to the spikes for a different hypothetical neuron. Spikes are binned in small intervals such that each interval contains at most one spike. This creates a discrete-time point process. PPF decodes the position with every 0 and 1. Example decoded positions in one dimension versus time are shown in dark blue and are updated with every 0 and 1. Hence any control and feedback rate below this high bin rate can be obtained. (**d**) The process of generating the controlled and feedback positions in PPF, FS-PPF and SS-PPF are shown for the same hypothetical spike train. Control rate is manipulated by adjusting how often the PPF's decoded position is sent to the prosthetic and used to control the task (controlled positions in light blue). Task success is assessed based on these controlled positions. Feedback rate is adjusted by changing the rate at which feedback of the controlled positions is provided to the subject (displayed positions in black). PPF consists of both a fast control and a fast feedback rate, FS-PPF consists of a fast control and a slow feedback rate, and SS-PPF consists of a slow control and a slow feedback rate. For illustration purposes, the slow rate is selected as one-third of the maximal bin rate in this figure. Blue arrows show which decoded positions are sent to the prosthetic over the forward path, and grey arrows show which controlled positions are displayed to the monkey over the feedback path.

We trained two adult male rhesus monkeys to perform a two-dimensional, self-paced centre-out reaching task with centre and target hold requirements under neural control (Fig. 1a, ‘Methods' section). The BMI was controlled by ensembles of multi-unit activity from the primary- and pre-motor cortices (15–33 units). At the beginning of each day, decoders were typically first trained using neural activity recorded during passive observation of cursor movements and were then adapted using closed-loop decoder adaptation (CLDA) techniques to achieve proficient control^16^,^31^,^32^ (see the ‘Methods' section and Supplementary Note 3).

To enable sensorimotor loop manipulations, we developed a closed-loop PPF BMI that directly modelled neural spikes as a series of random 0 and 1 events occurring in time. Point processes have been used in computational neuroscience to model the spikes in open-loop and offline studies, for example, to decode the position of a rat from hippocampal place cell activity^33^ or to decode hand movements from motor cortical activity offline^34^. These models also hold promise for BMI decoders^31^,^35^,^36^. Here we developed a PPF that was trained adaptively in closed loop using an optimal feedback control model of BMI^31^,^35^,^36^. The point process models a set of 1 and 0 events (that is, spikes or lack thereof) that occur in continuous time. Although in general these point process events are continuous time, they can be approximated as a discrete time point process by using small time bins containing at most one spike and generating a discrete time series of 0's and 1's (refs 33, 34, 37). As long as this bin-width is selected small enough to contain at most one spike, the discrete time process directly models the 0 and 1 time series of the spikes well^33^,^34^,^37^. Thus this point process modelling enabled the brain to control the BMI and receive feedback at an arbitrarily fast rate (that is, as fast as with every 0 and 1; Fig. 1c). To choose the bin width that allows for a good discrete time approximation to the continuous time 0 and 1 time series, we found that consecutive spikes from any unit rarely (less than 0.6% of the time) occurred within 5 ms of each other, and hence used a 5 ms bin width. The PPF therefore decoded the position at 200 Hz (180 Hz for monkey C). While the PPF updated the decoded cursor position with every 0 or 1 (Fig. 1c), we adjusted the control rate by changing how often this PPF output was sent to the prosthetic (Fig. 1b,d). Similarly, we manipulated the feedback rate by changing the rate at which the prosthetic's (that is, cursor's) position was updated on the subject's computer screen (Fig. 1b,d). These new experiments enabled the dissociation of the control and feedback rates (see Supplementary Note 1).

Since typical BMI systems run at around 10–20 Hz (refs 7, 9, 11, 12, 13, 14, 15, 19, 38), we used 10 Hz as our baseline pathway rate. We call a 10 Hz pathway (whether control or feedback) a slow pathway. To see the full effect of increasing the pathway rate, we compared with the maximal possible feedback rate enabled by the computer monitor (that is, 60 Hz) in both monkeys. We also compared with the maximal control rate, that is, equal to the PPF decoding rate of 200 Hz, in monkey J and to a 60 Hz control rate in monkey C (Supplementary Note 1). We refer to these as fast pathways in monkeys J and C, respectively. We then manipulated each of the control and feedback pathways to be either slow or fast. There were a total of four possible combinations of the control-feedback bidirectional pathways: slow–slow, slow–fast, fast–slow, fast–fast (Fig. 1d). The slow–fast pathway is equivalent to the slow–slow pathway since the cursor's position display cannot update at a rate faster than it is controlled. Thus we tested PPF decoders with the slow–slow, fast–slow and fast–fast control-feedback bidirectional pathway, referred to as SS-PPF, FS-PPF and PPF, respectively.

Each experimental day consisted of alternating blocks of decoder pairs to be compared, whose order was selected randomly (see Supplementary Table 1 for the number of paired blocks compared for each pair in each monkey). We use the rate of successful trial completion (‘success rate'), which takes into account both speed and task accuracy, as our main performance measure to assess the effects of control and feedback rates. In addition, we calculate percentage correct, reach time and movement error (see the ‘Methods' section). Performing direct BMI performance comparisons in the same animals, during the same task, and with the same recording quality ensures that variability due to extraneous factors like subject motivation and task proficiency, and differences in tasks and recording quality, which unavoidably exist across different labs, do not confound our results.

### Rapid control rates enhance neuroprosthetic control

**Figure 2:**
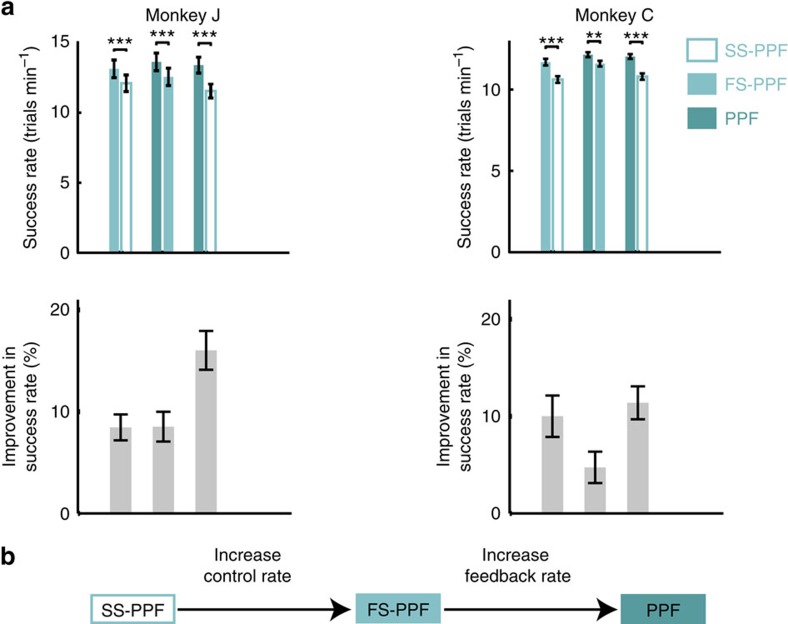
Rapid control and feedback rates enhance neuroprosthetic control. (**a**) Success rates for monkeys J and C using SS-PPF (white), FS-PPF (light blue) and PPF (dark blue). Each pair of bars indicates a paired comparison between two of the decoders and is obtained by averaging the performance of blocks that were run on the same days. Error bars indicate the s.e.m. Stars between the paired bars indicate a significant change, with one star indicating *P*<0.05, two stars indicating *P*<0.01 and three stars indicating *P*<0.001. The bottom panel shows the percentage improvement in the success rate for the paired bar comparisons in the top panel. Each grey improvement bar is calculated from the paired bars above it (that is, improvement of the left bar compared with the right bar in the paired bar above it). (**b**) SS-PPF has a slow control and a slow feedback rate, FS-PPF has a fast control rate but a slow feedback rate, and PPF has both fast control and fast feedback rates.

We first explored the influence of control rate in the feedforward pathway on neuroprosthetic control. To do so, we dissociated the effect of control and feedback rates and investigated the effect of increasing the control rate alone. We compared a slow-slow bidirectional pathway with a fast-slow one by having the monkeys control each of SS–PPF and FS–PPF decoders (Fig. 1d). This isolates the effect of increasing the control rate alone. Note that in FS–PPF, even though the cursor is controlled with every 0 and 1 event, feedback of its position is only provided to the subject every 100 ms. We find, surprisingly, that despite the slow feedback rate, allowing for control at a fast rate improves BMI performance. Both monkeys achieved a significantly higher success rate using FS–PPF (*P*<0.001, one-sided paired *t*-test, monkey C *n*=14, monkey J *n*=17; Fig. 2a, Supplementary Tables 1 and 2). The increased control rate also improved percentage correct significantly for both monkeys (*P*<0.001, one-sided paired *t*-test, monkey C *n*=14, monkey J *n*=17; Supplementary Fig. 1) and reduced movement error significantly for monkey J (*P*<0.03, one-sided paired *t*-test, *n*=17; Supplementary Fig. 1).

This result raises an important question as to why the increased control rate improves performance despite the fact that the increased control rate may be imperceptible to the subject when the feedback rate is the same. We found that the main reason for SS–PPF's degradation in success rate was a significant decrease in the ability to hold the target for the required hold period. In both monkeys, faster control rates did not impact reach times (SS–PPF versus FS–PPF reach times; *P*>0.23, two-sided paired *t*-test, monkey C *n*=14, monkey J *n*=17). The reach time similarity is also intuitively sound; just as a longer interval between consecutive cursor positions in SS–PPF could result in the cursor hitting the target later, it also could result in the cursor exiting the centre later (which is when we start counting the reach time). Hence the two effects cancel out. In contrast, in both monkeys, percentage correct was significantly decreased for SS–PPF (*P*<0.001, one-sided paired *t*-test, monkey C *n*=14, monkey J *n*=17). Since this change occurs with a fixed feedback rate, one hypothesis to explain this observation could be that subjects use a feedforward control strategy, which is more successful at higher control rates. Hence subjects' control may not be based purely on direct feedback of cursor positions. We performed detailed analyses (Supplementary Note 4) to examine the degradation in hold performance. These analyses show results that are consistent with a feedforward control hypothesis where a high-rate internal forward model is used to predict the cursor's time of entry in the target. Given the long visual feedback delays, it may be that the deceleration needed to satisfy the hold requirement is planned in a feedforward manner using a high-rate internal model. In this case, using a low-rate decoder (SS–PPF) the monkey will predict the time of entry incorrectly, which will result in higher hold errors in SS–PPF as observed in our data (see the ‘Discussions' section and Supplementary Note 4 for supporting analyses). The full examination and proof of such hypotheses requires careful perturbation experiments in future studies. The PPF BMI can facilitate such perturbations because it allows for manipulation of the sensorimotor pathway properties independent of the encoding model.

### Rapid feedback rates enhance neuroprosthetic control

We then explored the effect of the feedback rates. We compared a fast–slow pathway with a fast–fast one by having the monkeys control each of the FS–PPF and PPF decoders (Fig. 1d). We find that increasing the feedback rate improves BMI performance. Success rate significantly improved for both monkeys when feedback rate increased (*P*<0.005, one-sided paired *t*-test, monkey C *n*=17, monkey J *n*=17; Fig. 2a, Supplementary Table 2). The increased feedback rate also resulted in a significant reduction of reach time and movement error for both monkeys (*P*<0.02, one-sided paired *t*-test, monkey C *n*=17, monkey J *n*=17; Supplementary Fig. 1) and in a significant increase of percentage correct for monkey J (*P*<0.002, one-sided paired *t*-test, *n*=17; Supplementary Fig. 1). These results may suggest that the subjects utilize feedback information in their control strategy (see the ‘Discussion' section).

### Control and feedback rate enhancements combine

Finally, we explored the combination of fast feedback and fast control rates (Fig. 2b). In both monkeys, all performance measures significantly improved when increasing both control and feedback rates by controlling PPF as opposed to SS–PPF (*P*<0.002, one-sided paired *t*-test, monkey C *n*=16, monkey J *n*=22; Fig. 2a, Supplementary Fig. 1, Supplementary Table 2; see Supplementary Note 3 and Supplementary Fig. 1 for controls). Together, these results indicate that the brain's ability to control movement significantly depends on the control and feedback rates of the sensorimotor pathway. While a rate of around 10 Hz is common in current BMIs^9^,^11^,^14^,^15^,^19^, the brain has the capability to exploit both the faster rate of control and the faster rate of feedback for more proficient neuroprosthetic control.

### A new design direction for high-performance BMIs

The marked performance improvements with enhanced sensorimotor loop rates suggest a new direction for BMI design. The state-of-the-art KF is a rate-dependent decoder. It fundamentally limits the rate in the sensorimotor loop because of modelling assumptions. In particular, the KF is only optimal when the spike count within a bin is approximately Gaussian distributed. Hence KF-BMIs run at 10–20 Hz such that the bins are relatively large for the count to satisfy this assumption according to the central limit theorem^39^ (see Supplementary Notes 1 and 2). The performance of closed-loop KF BMIs has plateaued recently and new approaches based on the KF decoder have not been able to enhance neuroprosthetic designs^22^. Our results on rate effects suggest that the PPF, which provides a departure from KF-BMIs by modelling the spike 0 and 1 time-series and by allowing for arbitrary control and feedback rates, might significantly improve neuroprosthetic control over existing approaches.

**Figure 3:**
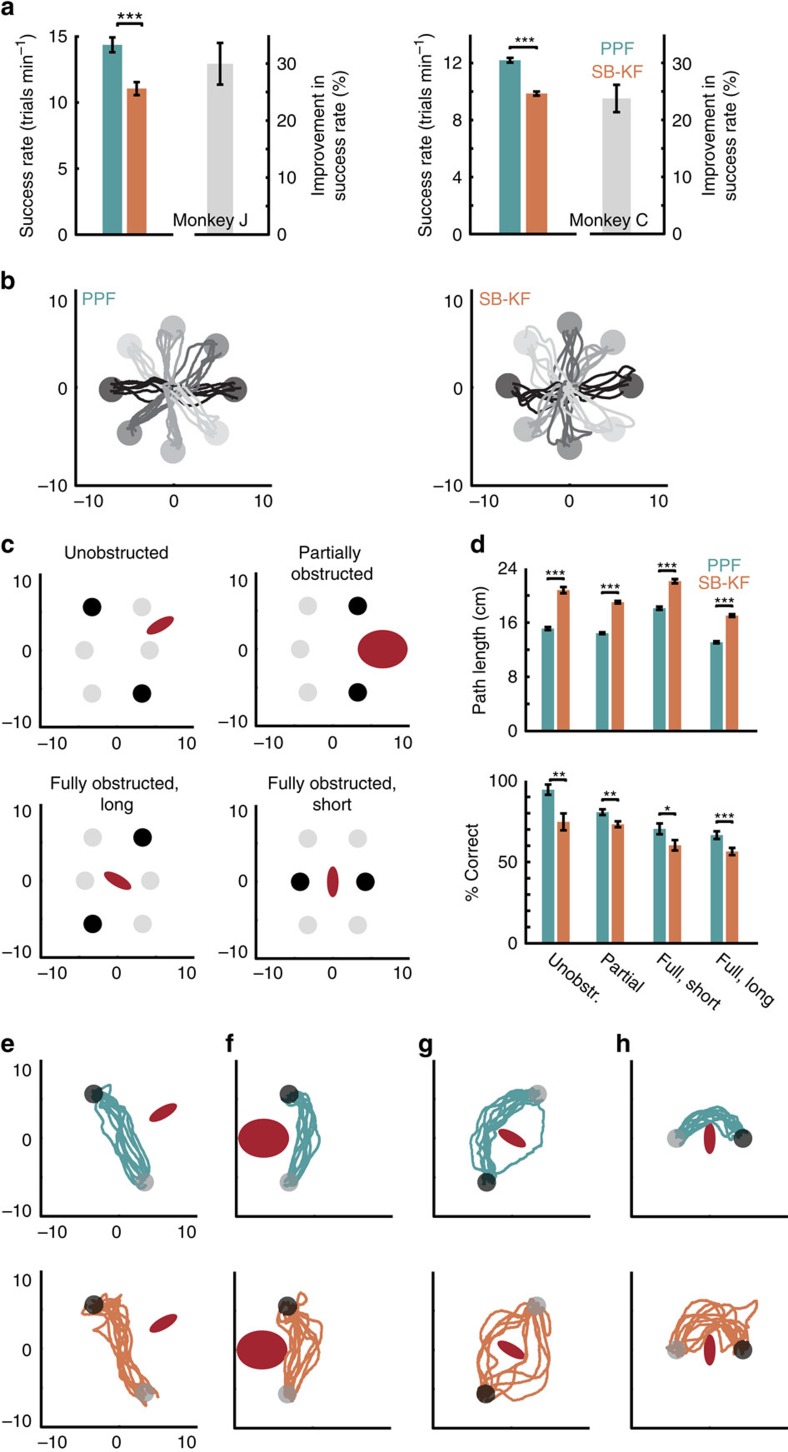
PPF enhances neuroprosthetic control compared with SB-KF in both monkeys. (**a**) Success rates using PPF and SB-KF on the same days and the corresponding percentage improvement in success rate when using PPF. Stars indicate a significant change with the same convention as in Fig. 2. (**b**) Random PPF and SB-KF centre-out trajectories. (**c**) Multi-curvature obstacle avoidance task. Each trial type consisted of a start target, an end target and an obstacle (red). The trials were constructed to include no obstruction, partial obstruction, full obstruction with a long distance between targets and full obstruction with a short distance between targets. Thus the obstacle avoidance task requires the monkey to perform movements of different lengths, locations and curvatures. (**d**) Comparison of path length and percentage correct (% correct) when monkey J used SB-KF or PPF for each of the four trial types in the obstacle avoidance task. Unobstr., unobstructed. (**e**–**h**) Sample trajectories (10 randomly selected per figure) using PPF (top) and SB-KF (bottom) for each of the four trial types.

We tested this hypothesis by comparing BMI performance with the PPF and a KF-based approach shown to provide current state-of-the-art performance^13^. We trained the KF decoder using SmoothBatch CLDA^16^ and an intention estimation technique^13^ that have been demonstrated to result in current state-of-the-art BMIs^13^,^16^ (‘Methods' section). We refer to this decoder as the SmoothBatch KF (SB-KF). We found that PPF markedly outperformed SB-KF in both monkeys (Fig. 3). Success rates using PPF were 30±3% and 24±2% higher than SB-KF in monkeys J and C, respectively (Fig. 3a, Supplementary Table 2; mean±s.e.m.; *P*<0.0001, one-sided paired *t*-test, monkey C *n*=20, monkey J *n*=7). Both movement error and reach time were significantly reduced when using PPF compared to SB-KF in both monkeys (Fig. 3b, Supplementary Fig. 2; *P*<0.002, one-sided paired *t*-test, monkey C *n*=20, monkey J *n*=7), and percentage correct was significantly increased in monkey J (Supplementary Fig. 2; *P*<0.005, one-sided paired *t*-test, *n*=7; see also control in Supplementary Fig. 2).

These performance improvements were robust both across time and assessment tasks. We performed 66 days of additional BMI experiments (33,468 trials total) with monkey J in which PPF and SB-KF were used on different days (Supplementary Fig. 3, Supplementary Table 2). Consistently, PPF improved all performance measures compared with SB-KF (*P*<10^−6^, one-sided *t*-test, 66 days), increasing success rate by 32±3% (mean±s.e.m.; *P*<10^−15^, one-sided *t*-test, 66 days). Thus PPF performance improvements were stable over months of recordings and were not affected by long-term learning of the two decoders. We also found that performance differences between the PPF and KF generalized to tasks beyond those used for CLDA training. We compared PPF with SB-KF in monkey J on a multi-curvature obstacle-avoidance task consisting of point-to-point reaches with an obstacle to be avoided during the movement (Fig. 3c). Consistently, PPF outperformed SB-KF in the obstacle task. The path lengths taken to avoid the obstacles were markedly longer when the monkey used SB-KF instead of PPF on every path type (Fig. 3d–h; *P*<10^−15^, one-sided *t*-test; 2,562 trials). On average, this difference was 30%. Moreover, percentage correct using the PPF was significantly higher on all path types (Fig. 3d; *P*<0.02, one-sided *t*-test; 2,562 trials) and success rate was on average 17% higher.

### Selecting the encoding model to enable high rates in BMIs

Across these comparisons, we found a consistently larger improvement between SB-KF and PPF than SS-PPF and PPF (Supplementary Tables 1 and 2; one-sided *t*-test; *P*<0.002). Beyond rate differences, the new PPF and KF also substantially differ in their neural encoding model (that is, the mathematical model that relates neural activity to intended velocity). While the PPF paradigm models the 0 and 1 time series of the spikes, the KF preprocesses the spikes first by turning them into counts and then models the counts. We hypothesized that the encoding model may also contribute to the observed performance improvements by more accurately modelling the spiking activity. We then used our PPF paradigm to isolate the effects of the encoding model on BMI performance improvement. We compared a PPF and a KF that were designed to have the same control and feedback rates. We first used a rate of 10 Hz (a slow–slow pathway) to compare the two models, that is, when KF has the benefit of larger bin-widths (because the KF is optimal if the spike counts in a bin have a Gaussian distribution, which is satisfied better at the larger bin widths; see the ‘Methods' section and Supplementary Notes 1 and 2). Even at this large bin-width, SS-PPF enabled higher success rates compared with SB-KF in both monkeys (Supplementary Fig. 4, Supplementary Table 2; *P*<0.006, one-sided paired *t*-test, monkey C *n*=11, monkey J *n*=14) and improved most other measures as well.

While the above comparison indicates the benefit of the point process modelling of spikes even at slow rates, a fundamental property of the point process model is that it allows the BMI to incorporate the higher control and feedback rates to enhance neuroprosthetic control. To allow for the higher control and feedback rates, the KF would also need to run at these high rates. We investigated whether the KF could enable high-rate control. We found that a high-rate KF resulted in a significant degradation of performance even compared with a low-rate version of itself (Supplementary Table 2; *P*<0.03, one-sided paired *t*-test, *n*=6; see also Supplementary Note 2 and control in Supplementary Fig. 4). These results indicate that the rate-independent PPF is better able to incorporate the high rate of control and feedback compared with rate-dependent decoders like the KF. Importantly, the PPF model facilitates high rate control. Taken together, by dissociating the effects of the increased control rate, increased feedback rate and the point process encoding model, our experiments indicate that each of these three factors contribute to more proficient neuroprosthetic control.

## Discussion

We developed a novel closed-loop PPF BMI and combined it with experiments in two macaque monkeys to dissociate the effects of two fundamental characteristics of the bidirectional sensorimotor pathway during neuroprosthetic control. We found that increasing the control rate, even when feedback rate was slower, improved control. Moreover, increasing the feedback rate further facilitated control. These results suggested the importance of high-rate sensorimotor pathways for BMI control. We proposed the PPF BMI, which enables these fast rates by modelling the 0 and 1 time series of the spikes using a point process. We compared the PPF with a state-of-the-art KF. PPF markedly outperformed the KF in both monkeys across various tasks, increasing success rate by 32% and 24% in monkeys J and C, respectively. Critically, our experiments demonstrate the key mechanisms facilitating these improvements—increased control rate, increased feedback rate and point process encoding models. Together, these findings highlight the importance of the sensorimotor pathway properties and the encoding model for neuroprosthetic design and control. By identifying factors that underlie performance improvements, our work provides insights for future developments in neuroprosthetic design.

Our work represents one of the first efforts to explore the effects of both sensorimotor control and feedback rates on neuroprosthetic control. Prior BMI studies^40^ have suggested that small bin-widths of 25–50 ms for the KF decoder may be beneficial for closed-loop control. However, changing the KF bin-width involves simultaneously changing all three factors explored in this study, that is, control rate, feedback rate and mathematical encoding model (Supplementary Note 1). Combined with our new perturbation experiments, the PPF allowed us to dissociate and examine the effect of each of these three factors because it is independent of rate and can run at any rate. Note that in principle, our perturbation experiments can be combined with any decoder that can run well at arbitrarily fast rates to manipulate the rate effects. However, the majority of BMI decoders used to date, like the KF, the population vector or the Wiener filter rely on a Gaussian assumption of the neural observations, which breaks downs at high rates (using small bins to count the spikes). This was confirmed here experimentally by the performance degradation of the KF at high rates (Results and Supplementary Fig. 4, Supplementary Table 2, see also the theoretical reason in Supplementary Note 2). Although it may also be possible to use a large sliding window in count-based decoders to compute the number of spikes and achieve a high rate, such a sliding window results in highly correlated observations because the spike counts are found using largely overlapping windows with the same spikes. Hence a sliding window approach does not adhere to the conditional independence assumption of observations in these decoders. Moreover, the amount of correlation introduced between the observations will be rate dependent (the higher the rate, the more the overlap and conditional dependence). Such a rate-dependent correlation could confound rate manipulation results. Finally, our results show that, regardless of the rate, a point process encoding model resulted in enhanced performance over the Gaussian encoding model used in previous decoders. Taken together, these commonly used BMI decoders are less useful for rate manipulations. Importantly, regardless of the choice of the decoder, this is the first study to dissociate and examine control and feedback rate effects in BMI.

We dissociated the effect of control and feedback rates for neuroprosthetic control. For natural motor control, psychophysics studies manipulating feedback intermittency^41^,^42^,^43^ and temporal delays^44^,^45^,^46^ have helped elucidate how visual feedback information is incorporated to guide motor control. However, in the natural motor system, control rates—dictated by the biophysical properties of muscle activation—are not readily amenable to manipulation making such exploration difficult. Also, dissociating the contribution of forward versus feedback processes to control output is believed to be difficult within psychophysics experiments^43^. The PPF BMI provides the opportunity to define the sensorimotor pathway properties and perform the perturbations required to examine their effects.

Our results demonstrate the brain's ability to incorporate feedback information and to control continuous movement of a prosthetic at rates higher than 10 Hz. Future studies testing a variety of feedback and control frequencies will be critical for fully characterizing the processing within the sensorimotor loop. Similar manipulations in electromyographic^47^,^48^ and kinematic interfaces^49^,^50^ could also be used to explore the influence of control rates across different levels within the motor system.

Our results may also shed light on the control strategies used by the primate brain in BMI. In natural motor control, many studies explored whether the brain uses feedforward or feedback control strategies^51^,^52^,^53^,^54^,^55^. It is unknown whether neuroprosthetic control uses similar mechanisms. Strikingly, we find that increasing the control rate, in the absence of an increased feedback rate, improves neuroprosthetic control. In this condition, the feedback pathway remains unchanged. Hence it is surprising that neuroprosthetic control is enhanced. One possible hypothesis could be that the brain uses a feedforward control strategy in performing the task (see Supplementary Note 4). Previous studies also suggest that the primate brain uses internal models during neuroprosthetic control^9^,^56^, which would have a critical role in feedforward and feedback control. Our results are consistent with a hypothesis that a high-rate internal forward model is involved in neuroprosthetic control. Specifically, we find that hold errors are significantly higher in slow control rate conditions compared with high control rate, suggesting the subjects cannot properly predict the cursor's time of entry into the target to successfully hold in the former case (see results and analyses in Supplementary Note 4). Given the long visual feedback delays and lack of proprioceptive information in BMIs, performing such feedforward prediction may be necessary for satisfying the hold condition at the target. Finally, neuroprosthetic control improved when the feedback rate was increased. This may imply the brain partially relies on feedback correction. Together, these results could suggest the hypothesis that the brain exploits a hybrid of feedforward and feedback strategies for neuroprosthetic control, consistent with current evidence from natural motor control^51^.

Although our data suggest possible control strategies, we emphasize that other alternative mechanisms may also explain our results, and more studies are needed. Since feedforward and feedback control strategies are closely tied to the properties of the sensorimotor loop, the PPF BMI provides a new tool by which to perform perturbation experiments and manipulate the pathway properties independent of other factors (such as the encoding model). This should allow the hypotheses about control strategies to be fully tested in future studies. This work can also be extended to analyse changes in neural activation patterns with sensorimotor loop manipulations. This may be a new, complementary avenue of investigation for understanding the relationships between neural network properties and the sensorimotor system^57^.

Our results have significant implications for neuroprosthetic technologies. The performance of KF-BMIs has remained unbeaten for several years. The PPF BMI markedly enhanced BMI performance over the current state-of-the-art KF-based approach. It also enabled a better understanding of the control processes underlying this improvement when combined with novel experiments. Identifying mechanisms underlying performance improvement provides principled design guidelines for future neuroprosthetic systems. These guidelines could be particularly useful for designing decoders for high-dimensional control tasks (for example, reach and grasp neuroprostheses) in future studies. The paradigm developed here will also allow for similar mechanistic study of pathway rates and encoding model effects in high-dimensional control, all of which may be particularly important as control and task complexities increase. Although two-dimensional control is itself a very active area within the BMI field that is essential for communication prostheses, exploring the performance of various BMI designs on higher dimensional tasks is an important future area of inquiry. In addition to setting the new state-of-the-art BMI performance, the PPF BMI may serve as a new scientific tool to understand fundamental mechanisms underlying neuroprosthetic control by providing a novel sensorimotor pathway whose rate characteristics can be readily manipulated.

## Methods

### Subjects and surgical procedures

Data were collected from two adult male rhesus macaques (*Macaca mulatta*) J and C. Subjects were chronically implanted with microwire electrode arrays (Teflon-coated tungsten electrodes, 35 μm diameter, 500 μm spacing; Innovative Neurophysiology, Durham, NC, USA) for neural recording. The arrays (128 electrodes; 8 × 16 configuration) were implanted bilaterally in the hand and arm representation areas of primary motor cortex (M1) and dorsal premotor cortex (PMd; Monkey J) and the primary sensory cortex (S1) and M1 (Monkey C), using stereotactic coordinates. All the procedures were conducted in compliance with the National Institutes of Health Guide for the Care and Use of Laboratory Animals and were approved by the University of California, Berkeley Institutional Animal Care and Use Committee.

### Electrophysiology

Neural activity was recorded from monkey J (C) using a 128- (256-) channel MAP (Omniplex) system (Plexon, Inc.). Multi-unit (monkey C) and channel-level activity (monkey J) was used for real-time BMI control. Multi-unit activity was sorted manually using online spike sorting software (Plexon, Inc.). Channel-level activity was defined by setting thresholds for each channel (5.5 s.d. from the mean signal amplitude) and using online sorting software to define unit templates that captured all incoming neural activity on the channel. Full details on channel-thresholding procedures are described elsewhere^19^. Over the course of experiments, BMI was controlled by 15–20 units in monkey J and by 33 units in monkey C.

### Behavioural task setup

Subjects were trained to perform behavioural tasks using arm movements. Following this training, they then transitioned to BMI control. Monkey J performed arm movement tasks in a two-dimensional environment with his right arm held within a KINARM exoskeleton (BKIN Technologies). The exoskeleton confined movements within the horizontal plane. Target information was projected onto a semi-silvered mirror parallel to the subject's arm movements (Fig. 1a); the end-point position of the subject's arm was presented as a dot on the screen co-localized with his hand. In BMI, monkey J's arm was removed from the exoskeleton, and placed within a primate chair. Changes in the colour of the cursor were also used to distinguish between BMI and arm control.

Monkey C performed a two-dimensional arm movement task by moving his right hand within a vertical plane in front of him. The hand position was tracked by a Phasespace Improv motion capture system (PhaseSpace Inc., San Leandro, CA, USA) and mapped to the position of a cursor displayed on a screen in front of the subject. Monkey C's arm was not restrained during BMI but was typically resting stationary by his side. Changes in the colour of the cursor and background were used to distinguish between BMI and arm control.

### Centre-out task

Subjects performed a self-paced delayed centre-out movement task with their arms (manual control) and under BMI-control. This task requires the animal to hold at the centre target for a specified period of time (the ‘delay'), before the go signal cues the animal to initiate the reach. Self-paced movements may be more representative of normal performance with a BMI and hence they were exploited in this study. Movements were made between a central target and eight peripheral targets uniformly spaced around a circle (Fig. 1a). Successful trials required a movement to and brief hold at the central target to initialize a trial, followed by a movement to and brief hold at the peripheral target. Failure to acquire the target within a specified window or to hold the cursor within the target for the duration of the hold period resulted in an error. To meet the hold requirement, subjects had to maintain the cursor within the target for the full hold-time duration upon entering it (that is, subjects had no opportunity to correct for exiting the target before the completion of the hold period, in contrast to some prior tasks).

Target directions were presented in pseudo-randomized blocks of eight targets. Monkey J (C) performed the task with circular targets of 1.2 (1.25) cm radii distributed about a circle of 13 (20) cm diameter. In BMI, hold times were 250 (216) ms and subjects had 3–10 s to complete the movement from central to peripheral target. Note that given the self-paced nature of movements, short reaction times were not reinforced. Despite this and even though the monkeys had 3–10 s to complete the task, they rarely took more than 2 s to complete the task in any condition (the mean reach time was approximately 1.4±0.3 s (mean±s.d.) across days and conditions).

### Obstacle-avoidance task

Monkey J also performed an obstacle-avoidance task under BMI control. This task required a point-to-point movement similar to the centre-out task (that is, movement to and brief hold at an initial target, followed by movement to and brief hold at a terminal target). In addition, on arrival at the initial target, an ‘obstacle' appeared on the screen. If the cursor entered the obstacle at any time during the movement to the terminal target, an error resulted and the trial was repeated.

Target positions and obstacle sizes and positions were selected to vary the amount of obstruction (from a straight-line path between targets), radius of curvature around the obstacles (via size of the obstacle or distance between the targets) and spatial locations of targets. Trials were constructed to include no obstruction, partial obstruction with low-curvature, full obstruction with a long distance between targets and full obstruction with a short distance between targets thus requiring a high curvature (Fig. 3c).

### Pair-wise decoder comparisons

Alternating blocks with random order were used on each day to compare a pair of decoders. The blocks were chosen either to be of equal time duration (typically 15 min, monkey J) or to consist of the same number of trials performed (typically 160 trials; monkey C). Since pair-wise comparisons between a given decoder (for example, PPF) with each of the other decoders (for example, SS-PPF and FS-PPF) could happen on different days, the performance bars corresponding to the same decoder could be slightly different among the different pair-wise comparisons. For example, the dark blue bars in Fig. 2a are not exactly the same. This is due to the behavioural and recording variability across days. This variability is the reason for us to do the decoder comparisons based on randomized blocks that were run on the same days (see Supplementary Table 1 for the number of paired-blocks compared).

### Behavioural metrics

BMI performance was quantified using both task-level and kinematic metrics. The main task metric used was success rate in each of the alternating blocks (number of successful trials completed per minute). We also calculated the trial percentage correct, also referred to as % correct (percentage of initiated trials resulting in success). In paired decoder comparisons using the alternating block structure, percentage correct was quantified by averaging over an equal number of trials in each of the alternating blocks, which was set to the number of trials in the block with the smaller number of trials (note that in monkey C paired blocks typically had the same number of trials and in monkey J blocks were of the same time duration). In monkey J and for the additional 66 experiments in which the decoders were run on separate days, success rate was calculated over the best 10 min and percentage correct was calculated over the best 100 consecutive trials per day for all decoders to minimize the effect of motivation variation. Movement kinematics were quantified using movement error and reach time metrics. Movement error for a given trial was defined as the average perpendicular deviation from a straight-line reach between the central and peripheral targets. Reach time was calculated as the time from leaving the central target to arriving at the peripheral target. Average reach time and movement error metrics for a session were calculated in the same manner as the percentage correct metric both for the alternating block experiments and for monkey J's experiments with decoders run on separate days. In the obstacle avoidance task, we use the total path length taken to avoid the obstacles as our measure of trajectory since movement error from the straight line path is no longer relevant.

### Closed-loop PPF BMI

We developed the PPF decoder, which controls the BMI continuously with every 0 and 1 spike event. The PPF thus allows us to keep the decoding model unchanged while changing the control and feedback rates independently to any value below the rate at which the spikes are binned to generate a 0 and 1 time series (200 Hz here; Fig. 1). To accomplish this, PPF models the spiking activity directly as a series of random 0 and 1 events occurring in continuous time using a point process. This continuous time point process can be approximated by a discrete time point process as long as the spikes are binned in small enough bins such that there is at most one spike per bin. Selecting the bin-width based on this criterion, the resulting discrete time point process can directly model the 0 and 1 time series of the spikes well^33^,^34^. Since consecutive spikes from any unit rarely occurred within 5 ms of each other (less than 0.6% of the time), we approximated this continuous time point process with a discrete time one by binning the spikes in Δ=5 ms intervals and creating a discrete time-series of 0's and 1's (refs 33, 34). We denote the binary spike event of the *c*-th neuron in the time interval [(*t*−1)Δ,*t*Δ], by 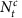. Assuming conditional independence between neurons, the point process observation model is given by


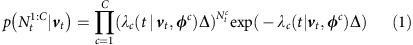


where 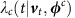 is the neuron's instantaneous firing rate, **v**_*t*_ is the velocity in the two dimensions and ***φ***^*c*^ are the parameters of the model to be estimated. We use a modified cosine tuning model of the motor cortex to write 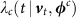 for each neuron as a log-linear function of velocity


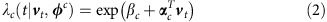


where 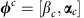 are the decoder parameters for neuron *c* that were estimated using closed-loop adaptation as described below.

Given the observation model in equation (1), we build a point process decoder for the kinematics^32^,^35^,^37^. The decoder consists of a prior model on the kinematic states and the point process observation model in equation (1). We define the state as 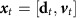 where the components represent position and velocity in the two dimensions. We build the prior on the kinematics as a general random-walk model that only enforces kinematic continuity


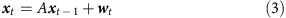


where *A* is the dynamic matrix and ***w***_*t*_ is a zero-mean white Gaussian state noise with covariance matrix *W*. We take *A* to be of the form


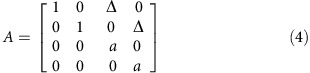


and *W* to be diagonal with non-zero diagonal entries for the velocity in the two dimensions, that is, *W*_3,3_=*W*_4,4_=*w*. We fit *w* and *a* to the monkey's manual cursor trajectory using maximum-likelihood methods^16^. For the log-linear instantaneous rate function in equation (2), the recursions of the PPF become^36^


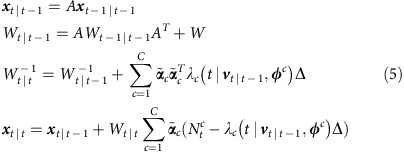


where 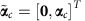
since we assume neurons are only tuned to velocity.

To perform our study, we first need to train the PPF decoder, find its parameters and then fix them to compare with a trained fixed KF. We train the decoder parameters 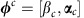 using closed-loop adaptation. As we described elsewhere^31^,^32^, to adapt these parameters, we first need to infer the intended velocity intention. Since the decoder parameters are far from optimal initially, the decoder output is a poor estimate of this intention. Hence we build an optimal feedback-control model of the brain^31^,^35^,^36^ to infer its velocity intention during adaptation. We then develop a second PPF that estimates the parameters in closed loop with every spike event and using the inferred intentions (see Supplementary Note 3 for details).

### SmoothBatch-KF BMI

We compare PPF to SB-KF. SB-KF has been described in detail previously^16^,^25^ (Supplementary Note 5). It is very similar to the ReFIT-KF^13^. However, instead of using a single long batch to refit the parameters, it uses consecutive batches of 60–90 s to refit the parameters, and smoothly averages these refitted parameters over time to obtain the updated decoder. SB-KF performs intention estimation by rotating the decoded velocity vector towards the target while keeping its magnitude unchanged, and by setting the velocity to zero while at the target^13^. Once KF was trained this way at the beginning of each day, it was fixed and used to conduct the present study. The prior model of SB-KF was related to that of the PPF in equation (3) using a simple calculation by observing that the state evolution in the PPF prior model after 20 time steps should be equivalent to the state evolution in the SB-KF prior model after a single time step (since the former's time step is 1/20 of the latter's). Hence parameters *a* and *w* of the prior model in equation (3) for KF and PPF are related as


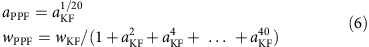


### Data availability

The data that support the findings of this study may be available on request from the corresponding author (J.M.C.).

## Additional information

**How to cite this article:** Shanechi, M. M. *et al*. Rapid control and feedback rates enhance neuroprosthetic control. *Nat. Commun.* **8,** 13825 doi: 10.1038/ncomms13825 (2017).

**Publisher's note:** Springer Nature remains neutral with regard to jurisdictional claims in published maps and institutional affiliations.

## Supplementary Material

Supplementary Information Supplementary Figures, Supplementary Tables, Supplementary Notes and Supplementary References
