# Supplementary material for: Rapid control and feedback rates enhance neuroprosthetic control
Source: Nat Commun. 2017 Jan 6;8:13825. doi: 10.1038/ncomms13825 (PMC5227098; doi:10.1038/ncomms13825)
Supplement: Supplementary Information — Supplementary Figures, Supplementary Tables, Supplementary Notes and Supplementary References [file ncomms13825-s1.pdf]

Supplementary Materials

Rapid Control and Feedback Rates Enhance Neuroprosthetic Control

Maryam M. Shanechi[1,2,5], Amy L. Orsborn[3,5], Helene G. Moorman[4,5], Suraj Gowda[2,5], Siddharth Dangi[2], Jose M. Carmena[2,3,4]

[1]Department of Electrical Engineering, Viterbi School of Engineering, University of Southern California, Los Angeles, CA 90089 USA
[2]Department of Electrical Engineering and Computer Sciences, University of California, Berkeley, Berkeley, CA 94720, USA
[3] UC Berkeley — UCSF Joint Graduate Program in Bioengineering
[4] Helen Wills Neuroscience Institute, University of California, Berkeley, Berkeley, CA 94720, USA
[5] authors contributed equally.

Correspondence should be addressed to M.M.S. (shanechi@usc.edu) and J.M.C. (jcarmena@berkeley.edu).

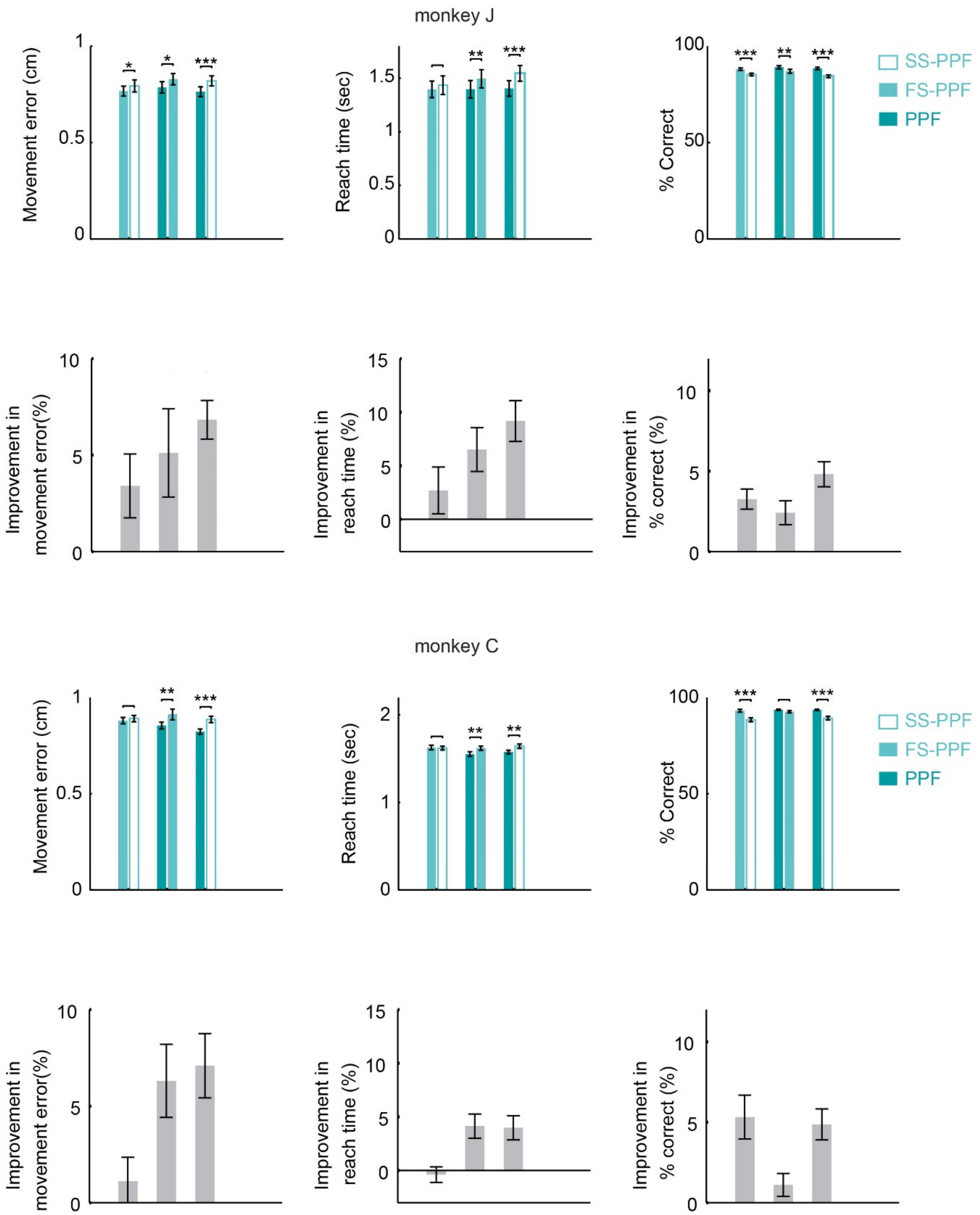

Supplementary Figure 1. Effect of rapid control and feedback rates on additional BMI measures for monkeys J and C. Figure has the same format as **Fig. 2A** but for movement error, reach time, and % correct measures. We also performed a control related to the effects of these

rates. We investigated whether training the point process model of the spikes on a slow-slow pathway improves the performance of SS-PPF (see **Supplementary Note 3**). We found that training the point process in the SS-PPF session did not improve the performance of SS-PPF. Success rate, movement error, reach time, and % correct did not change significantly when using SS-PPF trained in a SS-PPF session and SS-PPF trained in a PPF session (two-sided *t*-test, $P > 0.26$). In particular, success rate for SS-PPF whose point process model was trained in a SS-PPF session was 14.95 ± 0.95 compared to 15.65 ± 0.05 for SS-PPF whose point process model was trained in a normal PPF session (as is done in this work).

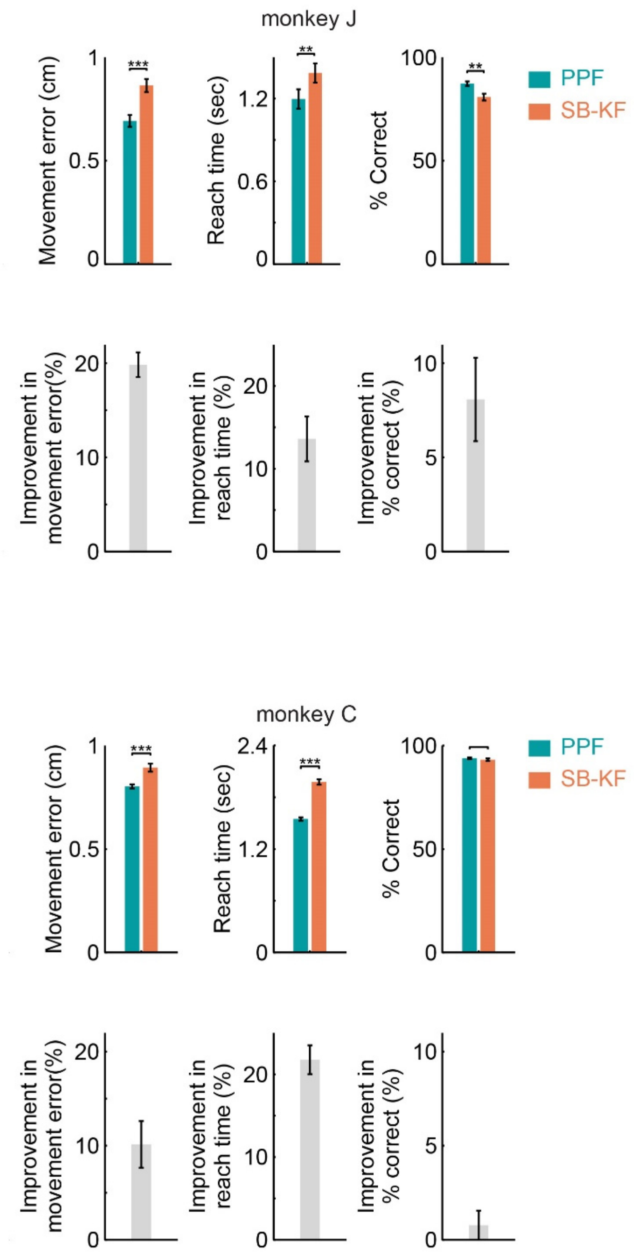

Supplementary Figure 2. Movement error, reach time, and % correct measures over the PPF and SB-KF blocks for monkeys J and C. Each paired bar is obtained by averaging the performance of blocks that were run on the same days. Stars indicate a significant change with the same convention as in **Fig. 2**. Bottom panels show the corresponding percent improvement in the measure when using PPF. As a control, we examined the effect of the method of intention estimation on SB-KF performance. The state-of-the-art KF performs intention estimation by

rotating the decoded velocity vector towards the target while keeping its magnitude unchanged, and by setting the velocity to zero while at the target[1]. We explored whether replacing this intention estimation method in the KF with the OFC method[2,3] could improve its performance. Closed-loop control results in monkey J showed that the success rate of the KF trained with OFC was not significantly different from the KF trained with the state-of-the-art intention estimation method (two-sided *t*-test, $P = 0.63$, 3739 trials).

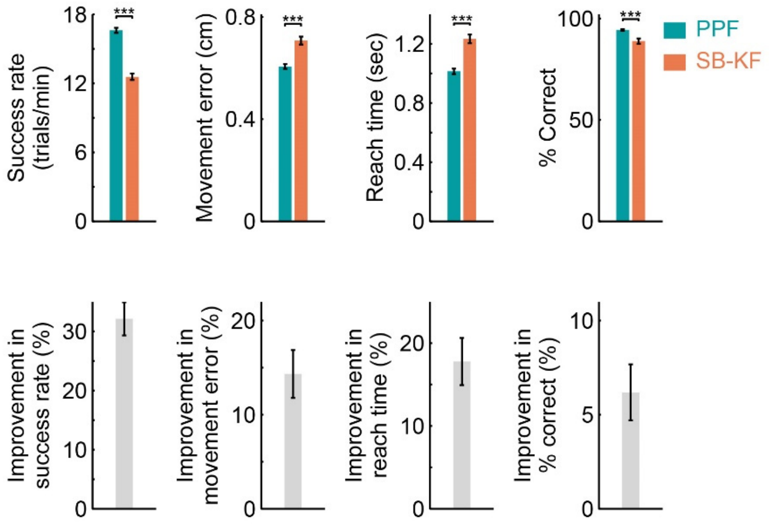

Supplementary Figure 3. PPF outperforms SB-KF in monkey J over 66 additional days of experiments in which PPF and SB-KF were run on separate days. Performance measures are shown in the *top* panels and the corresponding percent improvement in the measures when using PPF compared to SB-KF are shown in the *bottom* panels. Stars indicate a significant change with the same convention as in **Fig. 2**. These results are consistent with those in **Fig. 3**, indicating that PPF vs SB-KF performance improvement was stable over months of recordings and was not affected by long term learning of the two decoders.

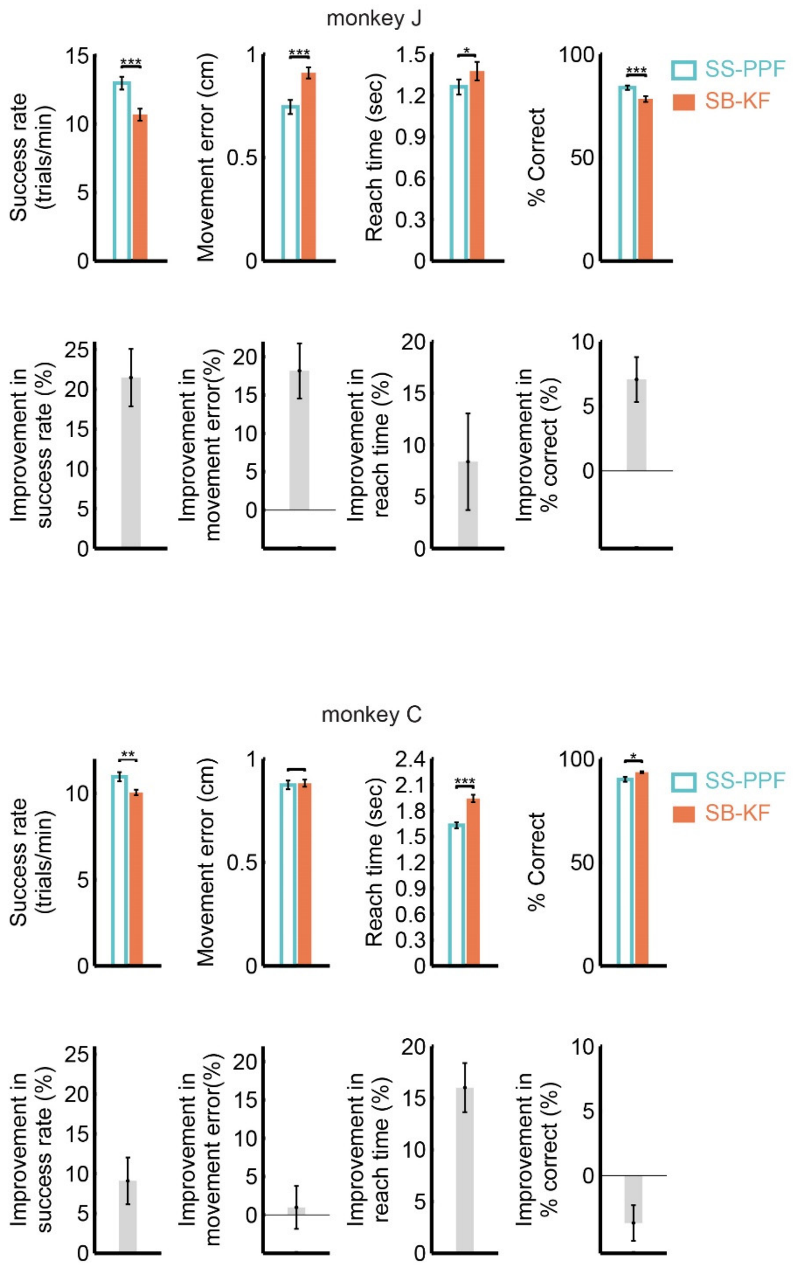

Supplementary Figure 4. Selecting the encoding model for high-rate BMIs. Success rates, movement errors, reach times, and % correct for SS-PPF (white) and SB-KF (orange). Figure convention is the same as **Fig. 2**. This figure shows that the point process encoding model outperforms the KF encoding model even at slow rates. To enable fast control and feedback rates, however, a mathematical encoding model should also enable running the BMI at fast rates

and should be able to model the spikes at fast rates. We thus tested the KF on a fast-fast pathway (i.e., using 5ms bins) and compared it to the typical KF over 6 sessions (see **Supplementary Notes 2, Supplementary Table 2**). This allowed us to explore the performance of the KF using 5ms bins to determine whether the KF could also provide the benefit of high rates to the subject. This is important to explore since the KF is only optimal when the neural observations are Gaussian distributed, which is satisfied better at larger bin-widths. At the fast rates with 5ms bins, the neural observations are 0/1 time-series and hence are not Gaussian distributed. Success rate was significantly higher in each monkey when using the KF with 100ms bins compared with the KF with 5ms bins (one-sided paired t-test, $P < 0.03$**, Supplementary Table 2**). Specifically, success rate was on average 70% and 19% higher using the KF with 100ms bins compared to the KF with 5ms bins in monkeys J and C, respectively. Hence increasing the pathway rate did not enable better control with the KF but degraded the quality of control likely as a result of the KF's Gaussian assumption on the binned spike counts being worse at small bin widths. This suggests that to take advantage of increased sensorimotor control and feedback rates in BMIs, a point process encoding model is more appropriate.

Supplementary Table 1. Number of blocks used in pair-wise comparisons for each monkey

	SS-PPF vs FS-PPF	FS-PPF vs PPF	SS-PPF vs PPF	SS-PPF vs SB-KF	PPF vs SB-KF
**Monkey C**	14	17	16	11	20
**Monkey J***	17	17	22	14	7

*Monkey J also performed an additional 66 days of BMI experiments with PPF and SB-KF during which the decoders were run on separate days. It also performed the obstacle task with PPF and SB-KF over a period spanning 6 days and consisting of 2562 total trials (1152 PPF trials and 1410 SB-KF trials).

Supplementary Table 2. Summary of performance comparisons

Here we summarize all comparisons performed in the study for the center-out task (see also **Supplementary Table 1**). For simplicity, we report a % *improvement* in the metric observed in the second decoder relative to the first. For example, a success rate improvement of 32% for SB-KF vs. PPF means success rates were 32% *higher* for the PPF compared to the SB-KF. In addition to the results for the randomized paired comparisons within day, we also show the result for the comparison of PPF to SB-KF in monkey J that was done across days, over an additional 66 day period, denoted by CO (across-day). Abbreviations: CO = center-out. Asterisks denote *p*-values where * = $p < 0.05$; ** = $p < 0.01$; *** = $p < 0.001$.

	Test	Decoders compared	Task	Success rate (trials/minute) J	Success rate (trials/minute) C
**Rate dissociations**	Control rate	SS-PPF vs. FS-PPF	CO	9±1***	10±2***
**Rate dissociations**	Feedback Rate	FS-PPF vs. PPF	CO	9±1***	5±1**
**Rate dissociations**	Control and feedback rate	SS-PPF vs. PPF	CO	16±2***	11±2***
**KF vs. PPF**	Encoding model	SB-KF vs. SS-PPF	CO	21±4***	9±3**
**KF vs. PPF**	New BMI paradigm vs. current state-of-the-art	SB-KF vs. PPF	CO (within-day)	30±3***	24±2***
**KF vs. PPF**			CO (across-day)	32±3***	—
**KF Controls**	Rate dependence	SB-KF 5ms vs. SB-KF 100ms	CO	70±11**	19±6*

Supplementary Note 1: Detailed Description and Discussion of Rate Manipulations

Real-Time Manipulation of the Control and Feedback Rates

Here we expand on how the control and feedback rate manipulations are performed. The process works as follows. We form the binary time-series of the spikes in real time by binning them in small bins containing at most 1 spike (taken to be 5ms here). We run the PPF decoder in real time on the binary time-series of a population of neurons as given in equation (5) in the 'Methods' section (see **Fig. 1c**). This generates a high-rate decoded trajectory based on the spike time-series in real time. Real-time manipulation of this high-rate decoded trajectory allows us to change the control and feedback rates. In the PPF (with high feedback and control rates), we let the monkey move the cursor with every decoded position; we also show every decoded cursor position to the monkey on the computer screen (**Fig. 1d**, first subfigure). To achieve a fast control rate and slow feedback rate, we let the monkey move the cursor with every decoded position, but do not show every decoded cursor position to the monkey. The monkey instead sees only one of every few controlled cursor positions on the computer screen depending on the feedback rate (**Fig. 1d**, second subfigure). This is equivalent to closing the eyes as one controls a movement and only intermittently opening them. To get the slow control rate, we don't let the monkey move the cursor with every decoded position. For example for a control rate of 10Hz, we only let the monkey move the cursor with every 20th decoded position (**Fig. 1d**, third subfigure); note that the decoding rate is still 200Hz (reflecting the 5ms spike bins). Hence for SS-PPF while every spike between the previous control time and the current control time (100ms interval) is processed to get the current controlled position, the subject cannot send control commands to the BMI at a high rate, regardless of how it generates the spikes or its control strategy. The subject can only send control commands to the BMI at a slow rate. Similarly, note that a 10Hz KF also processes all the spikes within a bin-width of 100ms. However, it only sends to the cursor one decoded position for all these spikes.

Dissociation and Manipulation of Control and Feedback Rates without Changing the Encoding Model – The Reasons for Using the PPF

Investigating the effect of control and feedback rates on neuroprosthetic control requires designing a careful BMI that exhibits two main properties: (1) performs at any rate while using

the same mathematical encoding model (so that model changes do not confound the results) (2) performs well at any rate (whether high or low). The PPF developed here exhibits these two properties. First, since it directly models the 0 and 1 time-series of the spiking activity, it uses the exact same mathematical encoding model regardless of the rate. It essentially runs at a high rate and can enable any lower rate by sending only one of every few decoded positions to the prosthetic or by showing only one of every few controlled position to the subject (while using a single encoding model for all rates). Second, the quality of modeling does not degrade when going to lower or higher rates for the point process filter because it directly models the 0 and 1 time-series of the spikes and provides different rates by real-time manipulation of the decoded positions as described above. Indeed in our experiments we see that the monkey can use the PPF at any rate.

Note that, in principle, any decoder that performs well at arbitrarily high rates could be combined with our novel manipulation experiments to investigate the rate effects. The majority of prior BMI decoders, such as the KF or the Wiener filter, however, are based on a Gaussian assumption on the neural observation model (e.g., spike counts or firing rates), which breaks down at high rates. This assumption will likely result in degraded performance of these decoders at high rates. We confirm this theoretical prediction here in our experiments for the KF decoder (see the 'Results' section and **Supplementary Fig. 4**), showing that a high-rate KF provides worse performance than a low-rate equivalent (see the 'Results' section, and **Supplementary Note 2, Supplementary Fig. 4, Supplementary Table 2**). Using the KF for the rate dissociation experiments performed in this study would require running the KF at high-rates and then performing our real-time manipulations to change the rates (without changing the encoding model). Because the KF is rate-dependent and better suited to lower rates (**Supplementary Table 2**), it would be sub-optimal to use the KF for these studies. More broadly, BMI decoders based on the Gaussian assumption of neural spiking observations are less useful for perturbation experiments to investigate rate effects.

Finally, changing the bin-width of the KF does not result in a BMI with either of the two properties required for rate manipulation, i.e., (1) the requirement to perform at any rate while using the same mathematical encoding model (so that the mathematical model does not confound

the results) (2) the requirement to perform well at any rate (whether high or low). First, the KF is a rate-dependent decoder and its mathematical encoding model changes for different bin-widths. For example, the number of spikes within 100 ms bins has a different distribution than the number of spikes within 200ms bins. These are two different binomial distributions (assuming static firing rates within the bin) and result in different Gaussian approximation models for the counts. Hence using a KF, any bin-width manipulation will involve both a rate change and a model change and cannot disambiguate the two (see also section below). Second, the quality of the KF model is strongly dependent on the bin-width or rate. KF is only optimal for Gaussian observations. The quality of the Gaussian approximation to the spike count is poor at high rates. Hence using the KF, the monkey's control could degrade for fast rates compared with slow rates as we have shown in our experiments (see the 'Results' section, **Supplementary Fig. 4, Supplementary Table 2**). This is explained in detail below.

Changing the Bin-Width of the Kalman Filter Cannot Dissociate the Rates

The following 4 factors all change simultaneously when changing the KF bin-width: 1) control rate; 2) feedback rate; 3) statistics of the spike count, i.e., the encoding model; 4) the approximation error associated with modeling the spike counts as Gaussian, i.e., goodness of the encoding model for which the KF is optimal (Note that the KF is only optimal for Gaussian observations).

1) Changing the KF bin-width changes the feedback and control rates simultaneously.
2) The encoding model also changes in the case of the KF when the bin-width changes. For example, if we use 100ms bins, the KF counts the number of spikes within 100 ms whereas if we use, say, 200ms bins, it counts the number of spikes within 200ms. These two counts have different statistics/distributions (the mean of the second count is larger, for example). These two counts could be modeled as two different binomial distributions. Also importantly, they result in two different Gaussian approximations to the count. For example, let's say we bin the 0/1 spikes in 5ms bins. With a 100ms KF bin-width and assuming static firing rates within the bin-width for illustration purposes, the spike count, y_t, is distributed according to a binomial distribution with parameters $N = \frac{100}{5} = 20, p = firing\ rate * 5ms$, i.e.,

$$p(y_t) = \binom{N}{y_t} p^{y_t}(1-p)^{N-y_t}$$

In the second case the KF count is a different binomial distribution with $N = \frac{200}{5} = 40$ and with the same p (assuming static firing rates within the bin-width for illustration purposes). Consequently, the Gaussian approximation to the count distribution in the first case has mean $20p$ and in the second case has mean $40p$ and the variances in the two cases are also different (these are only for illustration purposes). Hence the distribution of the observations and the Gaussian statistical model approximating them depends on the bin-width or equivalently the rate of the KF. This shows the dependence of the KF encoding model on bin-width.

3) The *quality* of the Gaussian approximation of the spike count distribution is also dependent on the bin-width or rate. The Gaussian approximation is more accurate at larger bin-widths. This can be explained using the central limit theorem—the sum of random variables in the limit of an infinite number of them becomes approximately Gaussian distributed. Hence larger bins mean closer agreement with a Gaussian observation model. Since Kalman filters are only optimal for Gaussian observations, the larger the bin-width, the better the KF model in describing the neural data. Hence changing the bin-width also changes the quality of the Gaussian approximation to the spike counts and consequently the performance of the KF due to the approximation error.

In summary, changing the bin-width in the KF cannot dissociate the effect of the control and feedback rates from each other, or from the encoding model or its goodness.

As also mentioned in the above subsection, using a high-rate Kalman filter results in poor performance and hence cannot be combined with our novel manipulations to change and study the rates.

Setting the fast feedback rate

While in principle any feedback rate can be selected, since our computer monitors refresh at 60Hz, the fastest possible feedback rate is effectively 60Hz. Indeed for monkey J, we sent the

cursor position to the monitor at the fast decoding rate of 200 Hz, but the monitor only displayed this at 60Hz.

Supplementary Note 2: Testing the KF at High Rates

Testing the SB-KF on a fast-fast pathway

While—to compare the encoding models—we also compared the PPF and SB-KF models at the slow 10Hz rate to give the KF the benefit of the larger bin-width (**Supplementary Fig. 4**), a more fundamental property of the PPF is that it enables fast control and feedback rates in the sensorimotor pathway. For the KF to also facilitate fast control and feedback rates, it needs to be able to run well at fast rates. Hence as a control we tested the KF on a fast-fast pathway and compared it to the typical KF over 6 sessions. Unlike the PPF model, the KF model changes for different pathway rates. We thus trained the fast-fast KF with 5ms bins using two methods. In the first indirect method, we first trained a typical 10Hz KF (i.e., with 100ms bins) using SmoothBatch CLDA. We then analytically obtained the parameters of the fast-fast KF from the 10Hz SB-KF as follows. The KF Gaussian neural observation model is given by,

$$\boldsymbol{y}_t = H[1, \boldsymbol{v}_t]^T + \boldsymbol{q}_t, \quad (1)$$

where $\boldsymbol{y}_t$ is the $C \times 1$ vector of neural spike counts at time t, H is the model parameter and $\boldsymbol{q}_t$ is the white Gaussian noise with covariance matrix Q. We first find H and Q using SmoothBatch and for 100ms bins. We then obtain the fast-fast SB-KF observation model parameters as

$$\begin{aligned} H_{FF} &= H/20 \\ Q_{FF} &= Q/20^2 \end{aligned} \quad (2)$$

where 20 is the ratio of the typical KF bin width to the fast-fast KF bin width (i.e., 100/5). Moreover, using the same argument as in the PPF case, the fast-fast KF system dynamics model was related to the typical KF dynamics model as given in equation (6) in the 'Methods' section. In the second direct method, we used SmoothBatch CLDA to adapt the KF in closed loop using 5ms bins. We found that the typical SB-KF using the 100ms bins significantly outperformed the SB-KF using the 5ms bins regardless of the method of adaptation (**Supplementary Fig. 4, Supplementary Table 2**).

KF Performance for Spiking Observations

In this work we showed that regardless of the control or feedback rates, the PPF enables more proficient BMI control compared with the KF. Moreover, we showed that a high-rate KF performance degraded compared with a low-rate KF (see 'Results' section, **Supplementary Fig.**

4, Supplementary Table 2). Here we expand on the theoretical reason why the PPF model has an advantage, especially at high rates.

For the KF to be an optimal decoder, neural observations would need to be Gaussian distributed[4]. However, spiking activity, i.e., a time-series of 0's and 1's or a count calculated from these 0's and 1's are neither continuous-valued nor Gaussian. In contrast, the point process filter directly models and operates on the 0/1 time-series of spikes and hence is the approximately optimal filter for spiking activity[5–7]. When neural observations are not continuous-valued or are not approximately Gaussian, there is no guarantee that a Kalman filter would perform well.

In the case of fast rates, the neural observations are far from Gaussian. Indeed they are not continuous-valued. These observations are spikes and hence discrete-valued taking values 0 or 1. Hence the modeling assumptions needed for optimality in the KF are violated. This could explain why the KF does much worse at fast rates compared to PPF or to a slow-rate KF (**Supplementary Table 2**). There have been various studies suggesting this point about point process vs. Gaussian models in open-loop applications for example when decoding the hippocampal place cell activity offline collected from a rat running in a maze[6]. Our closed-loop BMI results provide experimental evidence that even in a closed-loop scenario, when the animal is in the loop, the direct modeling of spikes by a point process results in better closed-loop BMI performance (**Supplementary Table 2**).

In the case of the slow rate, the KF again did worse than the PPF (**Supplementary Table 2**). However, it did better than the KF with the fast rate. What can explain this is that the neural observations in larger 100ms bins are no longer only 0 or 1 but rather they are equal to the total number of spikes occurring in a 100ms bin. Hence the distribution of these counts is closer to a Gaussian distribution, which is the necessary condition for KF optimality. This can be explained based on the central limit theorem—the sum of random variables in the limit of an infinite number of them becomes approximately Gaussian distributed. However, despite the Gaussian approximation being better in this case, the number of spikes within a 100ms bin is still not fully Gaussian-distributed. The point process filter operates on the underlying signal—the spikes—and models their 0 and 1 time-series regardless of the rate used in the BMI.

Supplementary Note 3: Closed-Loop Model Adaptation and Training

Training the PPF decoder using OFC-PPF closed-loop adaptation

Before conducing the present study, we need to train both the KF and the PPF decoders at the beginning of each day, and then fix them to study their steady-state performance in continuous closed-loop BMI control across various rate conditions and tasks. Similar to the KF, we also train the PPF adaptively using CLDA. We denote the sequence of kinematic states by $\boldsymbol{x}_0, \cdots, \boldsymbol{x}_t$ and assume that they evolve in the linear dynamical system

$$\boldsymbol{x}_t = A\boldsymbol{x}_{t-1} + B\boldsymbol{u}_t + \boldsymbol{w}_t, \quad (3)$$

with parameters A and B. Here $\boldsymbol{u}_t$ is the control command at time t that the brain issues. We assume that the brain controls the velocity of movement and hence $\boldsymbol{u}_t$ is a velocity command in the two dimensions (i.e., $\boldsymbol{u}_t$ is 2×1) and $B_{2,2} = \Delta$, $B_{4,2} = \Delta$ and $B_{i,j} = 0$ for all other i, j where $B_{i,j}$ indicates the (i,j)th element of B. Note that (3) is the dynamics model we use for intention estimation, which is implicitly assumed to be a forward model of the movement that the brain has acquired[8,9]. This could thus be different from equation (3) in the 'Methods' section, which is the prior model used in the filter for decoding purposes and is thus selected to be as general as possible.

To predict the brain's intended velocity command, $\boldsymbol{u}_t$, we quantify the goal of the center-out task in a cost function. We then model the brain as an optimal feedback controller[2,10,11] (OFC) that minimizes this cost function over the choices of $\boldsymbol{u}_t$. We use an infinite-horizon quadratic cost function to quantify the task goal. For the center-out task, the goal is to take the cursor to the target location, stop there, and use minimum control effort to do so. We hence form the cost function as

$$J = \sum_{t=1}^{\infty} \|\boldsymbol{d}_t - \boldsymbol{d}^*\|^2 + w_r\|\boldsymbol{u}_t\|^2, \quad (4)$$

where $\boldsymbol{d}^*$ is the target location. We also assume that the monkey perfectly observes the current position of the cursor on the screen, which is the decoded position denoted by $\boldsymbol{x}_{t|t}$ at each time. Given the linear state-space model in (3) and the quadratic cost function in (4), the optimal $\boldsymbol{u}_t$

that minimizes the expected cost is given by the standard linear-quadratic Gaussian (LQG) solution. We hence find the velocity intention at each time as

$$\boldsymbol{u}_t = -L(\boldsymbol{x}_{t|t} - \boldsymbol{x}^*), \tag{5}$$

where $\boldsymbol{x}^* = [\boldsymbol{d}^*, \boldsymbol{0}]^T$ is the target state for position and velocity, and L is the steady-state solution to the algebraic Riccati equation found recursively and offline[2,3,10].

Given the inferred velocity intention $\boldsymbol{u}_t$ and the observed neural activity $N_{1:t}^{1:C}$ we now need to estimate the parameters of the point process model in closed loop. To do so, we design a spike-by-spike adaptation algorithm in which the point process parameters are updated with every 0 and 1 event[2,3]. This is done by developing a point process decoder for the parameters. The states in the parameter decoder are the parameters $\boldsymbol{\phi}^c$. We build the prior model of the parameters as a random-walk model given by

$$\boldsymbol{\phi}_t^c = \boldsymbol{\phi}_{t-1}^c + \boldsymbol{z}_{t-1}, \tag{6}$$

where $\boldsymbol{z}_{t-1}$ is white Gaussian noise. This model indicates that parameters evolve continuously. Using equation (1) in the 'Methods' section, the observation model is given by

$$p(N_{1:t}^{1:C}|\boldsymbol{u}_{1:t}) = \prod_{\tau=1}^{t}\prod_{c=1}^{C}(\lambda_c(\tau|\boldsymbol{u}_\tau, \boldsymbol{\phi}_\tau^c)\Delta)^{N_\tau^c} \exp(-\lambda_c(\tau|\boldsymbol{u}_\tau, \boldsymbol{\phi}_\tau^c)\Delta), \tag{7}$$

Note that $\boldsymbol{u}_{1:t}$ are the inferred velocity intentions given by (5). We can hence obtain the recursions of the point process decoder for the parameters similar to equation (5) in the 'Methods' section by reversing the roles of $\boldsymbol{\phi}_\tau^c$ and $\boldsymbol{u}_\tau$[2,3].

Adapting the point process model on a slow-slow pathway vs. a fast-fast pathway

The point process encoding model is trained using the PPF decoder on a fast-fast pathway. As a control, we investigated whether training the point process model of the spikes on a slow-slow pathway improves the performance of SS-PPF. To do so, we performed experiments in which we adapted the point process model as the monkey controlled the SS-PPF as opposed to the normal PPF. We then constructed two SS-PPF decoders. One used the point process trained in the SS-PPF session and the other used the point process trained in the normal PPF session. We found that training the point process in the SS-PPF session did not improve the performance of SS-PPF (control in **Supplementary Fig. 1**).

Supplementary Note 4: Examining a plausible mechanism that may underlie the improvement in FS-PPF vs. SS-PPF

We attempted to find a plausible explanation for the observation that holds are significantly degraded with SS-PPF compared to FS-PPF. This was the main reason for degradation of performance (i.e., success rate) in SS-PPF. The hold period started once the cursor entered the target. Hence the monkey would need to know when the cursor enters the target to satisfy the hold period. However, given the long visual feedback delays (motor-cortical latencies to visual stimuli) of about 120-140ms[12,13], the monkey cannot wait for the visual feedback of the cursor position—once it enters the target—to start planning the hold, e.g., to start a deceleration strategy. This visual feedback will come with a delay of ~120-140ms, which may be too late to decelerate and initiate a hold process (depending on the cursor speed). Hence we propose that to perform the holds, the monkey may have to predict in a feedforward manner when the cursor would enter the target. A plausible way to perform this prediction would be to use an internal forward model of the BMI movement. If the decoder model matches the monkey's internal forward model, then the prediction of the entry time is accurate and the monkey will initiate and plan the hold process according to the correct entry time and succeed.

One possible hypothesis could be that the monkey's strategy in performing a hold is closer to feedforward prediction using a high-rate forward dynamic model compared with a low-rate forward dynamic model, given the better control under FS-PPF. To examine this hypothesis, we performed the following analyses in the SS-PPF condition. We computed what time the monkey would predict the cursor to enter the target according to a high-rate forward model (matched to the high-rate decoder). We then computed whether the monkey satisfied the hold requirement starting from this predicted entry time (even though in the *actual* SS-PPF trial, he may not be satisfying the hold). If these 'adjusted' hold times in the SS-PPF trials satisfy the hold requirement, then this provides some evidence consistent with our hypothesized control mechanism. That is, it would suggest the monkey's strategy is close to a feedforward strategy based on a high-rate forward model (as compared to a low rate forward model for example). Importantly, we would expect the 'adjusted' SS-PPF performance to be similar to the FS-PPF condition, i.e., for the difference to become smaller.

Thus we calculated what the percent correct (i.e., hold success) would be if we start counting the hold time starting from the time the monkey would predict the cursor to enter the target using a high-rate forward model. To do this, we found what the time of target entry would have been if there was no real-time subsampling for the controlled trajectory. This entry time can be readily computed post-hoc by using the original high-rate neural spike commands and the corresponding decoded trajectory (which was subsampled in real-time in the slow control-rate condition). Once this predicted entry time was computed for each trial, we calculated an 'adjusted' hold time that starts counting from this forward-predicted time. We tested whether this 'adjusted' hold time satisfies the hold requirement even if the SS-PPF trajectory does not ('adjusted' hold time= time the cursor exited the target - the predicted entry time from monkey's perspective). This allowed us to calculate the 'adjusted' success rate.

We found that the 'adjusted' SS-PPF percent correct was significantly higher than the SS-PPF percent correct. In particular, percent correct improved by 8% and 7% for monkeys J and C, respectively ($P<10^{-4}$, one-sided paired *t*-test, monkey J n=38, monkey C n=19). The adjusted reach time did not improve as expected ($P>0.1$, two-sided paired *t*-test, monkey J n=38, monkey C n=19). Importantly, once we made this adjustment, the difference between the performance (success rate) in FS-PPF and 'adjusted' SS-PPF almost vanished (two-sided paired *t*-test, $P>0.45$ in monkey J n=17 and $P>0.06$ in monkey C n=14).

One interesting point to note is that while the subject does not necessarily need to be trying to stay within the target boundaries for just the required hold period and not longer, our analyses indicate that the subject may indeed be doing so. In particular, the subject may have optimized its control strategy to receive reward by staying inside the target boundaries just as long as the required hold time and not longer. That seems to be the reason why if the entry time is just a bit off from the predicted time, the error rate goes up.

Finally, since the animal is not cued to the particular condition (i.e., SS-PPF vs. FS-PPF vs. PPF), it is possible that he is not aware of the condition, at least initially. The above argument does not require the monkey's strategy to change or the monkey to be aware of the condition.

Even in the absence of a strategy change, what our results may indicate are that (i) the hold precision (percent correct) significantly drops in the SS-PPF and (ii) this drop is because no matter what the monkey's control strategy is, the high-rate decoder better matches it. However, given that the error rate significantly increases in the SS-PPF case, it is also possible that the monkey does recognize this condition based on the reward feedback and hence changes its strategy. We examined whether the success rate changed between the first and second halves of the SS-PPF blocks and did not observe a significant difference ($P>0.21$; two-sided paired t-test, monkey J n=38, monkey C n=19). This may indicate that either the control strategy doesn't change between SS-PPF and FS-PPF or that it is very hard for an alternative strategy to improve performance in the SS-PPF case (given the high-rate internal forward model that may be used for example).

Overall, the results of these analyses are consistent with the hypothesis that the target holds may be performed in a feedforward manner in BMI, and that, assuming a feedforward control strategy in BMI, the monkey's internal forward model is closer to a high-rate model compared with a low-rate model. The high-rate dynamic decoder may better match the monkey's own internal model, thereby allowing the monkey to perform more accurate feedforward prediction and control, and thus result in higher performance (see Discussion). The full examination of this hypothesis requires careful perturbation experiments in future studies, which the PPF BMI can enable.

Supplementary Note 5: KF and PPF as recursive Bayesian estimators

KF and PPF are both examples of a recursive Bayesian estimator. A recursive Bayesian estimator consists of two models: a prior model on the time-sequence of kinematics states and an observation model relating the neural activity to these kinematic states. The mathematical distinction between the KF and the PPF is the observation model. In KF-BMIs, the observation model is constructed for spike *counts* (typically estimated using 50-100ms bins). The observation model is assumed to be linear and Gaussian, i.e., spike counts are assumed to be Gaussian-distributed and linearly related to the kinematics. In the PPF-BMI, the observation model is constructed for the 0 and 1 time-series of spike *events*. The observation model is a point process whose instantaneous rate function is assumed to be log-linearly related to kinematics.

The recursive nature of the recursive Bayesian estimator allows it to run efficiently and in real time. Hence both KF and PPF are real-time and well-suited for BMI applications.

The prior model allows a recursive Bayesian decoder to be predictive. This means that a recursive Bayesian estimator (whether KF or PPF), can use the prior state evolution model and the current estimate of the kinematic state to predict the next kinematic state. Both PPF and KF are predictive.

Finally, both PPF and KF can accommodate adaptation in their training procedure or in response to non-stationarity. Adaptive methods to train both KF decoders and PPF decoders have been developed. For example, batch-based methods[1,14,15] or continuous adaptation methods[16] have been developed to adaptively train the KF. We have also developed an adaptive method to train the PPF[2,3]. Hence in terms of being adaptive, both KF and PPF have the option of incorporating adaptation in their design and need not be fixed in time. In this study, at the beginning of each day, we used adaptive training for both the KF and PPF using established methods. Performance, however, was compared for fixed trained models, which were found to yield highly stable performance within a session (consistent with many previous reports[1,14–16]). This comparison approach avoids potential confounds of ongoing adaptation and algorithmic convergence, allowing for more direct assessment of decoder differences in steady-state continuous closed-loop BMI control.

Supplementary References

1. Gilja, V. *et al.* A High-Performance Neural Prosthesis Enabled by Control Algorithm Design. *Nat. Neurosci.* **15**, 1752–1757 (2012).
2. Shanechi, M. M., Orsborn, A. L. & Carmena, J. M. Robust Brain-Machine Interface Design Using Optimal Feedback Control Modeling and Adaptive Point Process Filtering. *PLoS Comput. Biol.* **12**, e1004730 (2016).
3. Shanechi, M. M. & Carmena, J. M. Optimal Feedback-Controlled Point Process Decoder for Adaptation and Assisted Training in Brain-Machine Interfaces. *Proc. IEEE EMBS Conference on Neural Engineering (NER)* (2013).
4. Anderson, B. D. O. & Moore, J. B. *Optimal Filtering*. (Dover Publications: New York, 2005).
5. Brown, E. N., Barbieri, R., Ventura, V., Kass, R. & Frank, L. The time-rescaling theorem and its application to neural spike train data analysis. *Neural Comput.* **14**, 325–346 (2001).
6. Brown, E. N., Frank, L. M., Tang, D., Quirk, M. C. & Wilson, M. A. A statistical paradigm for neural spike train decoding applied to position prediction from ensemble firing patterns of rat hippocampal place cells. *J. Neurosci.* **18**, 7411–7425 (1998).
7. Truccolo, W., Eden, U. T., Fellows, M. R., Donoghue, J. P. & Brown, E. N. A point process framework for relating neural spiking activity to spiking history, neural ensemble, and extrinsic covariate effects. *J. Neurophysiol.* **93**, 1074–1089 (2005).
8. Golub, M. D., Yu, B. M. & Chase, S. M. Internal Models Engaged by Brain-computer Interface Control. *IEEE EMBS* (2012).
9. Shadmehr, R. & Krakauer, J. W. A computational neuroanatomy for motor control. *Exp. Brain Res.* **185**, 359–381 (2008).
10. Shanechi, M. M., Wornell, G. W., Williams, Z. M. & Brown, E. N. Feedback-controlled parallel point process filter for estimation of goal-directed movements from neural signals. *IEEE Trans. Neural Syst. Rehabil. Eng.* **21**, 129–140 (2013).
11. Shanechi, M. M. *et al.* A Real-Time Brain-Machine Interface Combining Motor Target and Trajectory Intent Using an Optimal Feedback Control Design. *PLOS ONE* **8**, e59049 (2013).
12. Georgopoulos, A. P., Kalaska, J. F., Caminiti, R. & Massey, J. T. On the relations between the direction of two-dimensional arm movement and cell discharge in primate motor cortex. *J. Neurosci.* **2**, 1527–1537 (1982).
13. Schwartz, A., Kettner, R. & Georgopoulos, A. Primate motor cortex and free arm movements to visual targets in three- dimensional space. I. Relations between single cell discharge and direction of movement. *J. Neurosci.* **8**, 2913–2927 (1988).
14. Orsborn, A. L., Dangi, S., Moorman, H. G. & Carmena, J. M. Closed-Loop Decoder Adaptation on Intermediate Time-Scales Facilitates Rapid BMI Performance Improvements Independent of Decoder Initialization Conditions. *IEEE Trans. Neural Syst. Rehabil. Eng.* **20**, 468–477 (2012).
15. Orsborn, A. L. *et al.* Closed-loop decoder adaptation shapes neural plasticity for skillful neuroprosthetic control. *Neuron* **82**, 1380–1392 (2014).
16. Dangi, S. *et al.* Continuous Closed-Loop Decoder Adaptation with a Recursive Maximum Likelihood Algorithm Allows for Rapid Performance Acquisition in Brain-Machine Interfaces. *Neural Comput.* **26**, 1811–1839 (2014).
